# Supplementary material for: Repurposed Drugs That Activate Autophagy in Filarial Worms Act as Effective Macrofilaricides
Source: Pharmaceutics. 2024 Feb 9;16(2):256. doi: 10.3390/pharmaceutics16020256 (PMC10891619; doi:10.3390/pharmaceutics16020256)
Supplement: Supplementary file 1 [file pharmaceutics-16-00256-s001.zip › Table S1.pdf]

Table S1. Autophagy inducing compounds used in this study.

Name	Catalog number	Company	Purity	Structure
FK866	ALX-270-501	Enzo Life Sciences, Farmingdale NY	≥98%	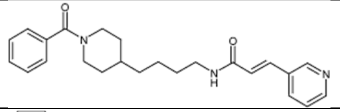
TTFA (2-Thenoyltrifluoroacetone)	15517	Cayman Chemical Company, Ann Arbor MI	≥98%	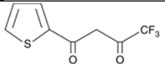
Imatinib	ALX-270-492	Enzo Life Sciences, Farmingdale NY	≥98%	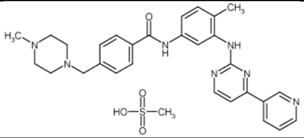
Metformin-HCl	ALX-270-432	Enzo Life Sciences, Farmingdale NY	≥97%	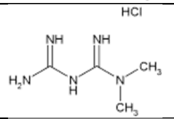
Minoxidil	M4145	Sigma, St. Louis MO	≥99%	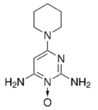
PI-103	S1038	Selleckchem, Houston TX	≥99.59%	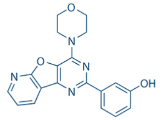
Clonidine HCl	S2458	Selleckchem, Houston TX	≥99.97%	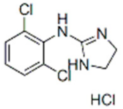
Niclosamide	481909	Sigma, St. Louis MO	≥97%	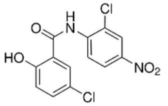
Rottlerin	ALX-350-075	Enzo Life Sciences, Farmingdale NY	≥98%	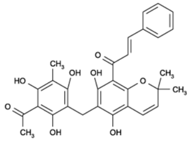
